# LMO3 downregulation in PCa: A prospective biomarker associated with immune infiltration

**DOI:** 10.3389/fgene.2022.945151

**Published:** 2022-09-19

**Authors:** Wenchao Xu, Taotao Sun, Jiaxin Wang, Hao Li, Bingliang Chen, Yingjian Zhou, Tao Wang, Shaogang Wang, Jihong Liu, Hongyang Jiang

**Affiliations:** ^1^ Department of Urology, Tongji Hospital, Tongji Medical College, Huazhong University of Science and Technology, Wuhan, China; ^2^ Institute of Urology, Tongji Hospital, Tongji Medical College, Huazhong University of Science and Technology, Wuhan, China

**Keywords:** LMO3, prostate cancer, biomarker, immune infiltration, immunotherapy

## Abstract

Prostate cancer is the third leading cause of new cancer cases and the second most common tumor type in men globally. LMO3 has been stated to play a vital role in some cancers; however, the prognostic value of LMO3 in PCa remains vague. Here, we utilized various web databases to elucidate in detail the prognostic value and molecular functions of LMO3 in PCa. LMO3 expression was significantly decreased in PCa. Low LMO3 expression was associated with gender, age, and TNM grade and predicted a poor prognosis in PCa patients. Functional enrichment analysis suggested that LMO3 is engaged in the extracellular matrix and immune response. Moreover, LMO3 was positively correlated with immune infiltration levels and numerous immune markers. LMO3 may function as a prospective biomarker of immune infiltration in PCa.

## 1 Introduction

Prostate cancer (PCa) is the third leading cause of new cancer cases and the second most common tumor type in men around the world (Sung et al., 2021). Due to refractoriness to androgen deprivation therapy, the burden of PCa on health and the economy remains critical (Moreira et al., 2017; Force, 2018). PCa is characterized by a remarkable heterogeneity, in which some patients experience an indolent course and only need active surveillance, whereas others progress rapidly and require early comprehensive treatment (Sternberg et al., 2020). Therefore, this raises an urgent need for identifying reliable prognostic biomarkers that can refine the risk evaluation for long-term survival (Bhanvadia et al., 2018). However, the pathogenesis of PCa is understudied, and contributing mechanisms are unclear (Haffner et al., 2020). Therefore, the identification of novel significant markers is critical for the diagnosis and prognosis of PCa.

The LIM-domain-only (LMO) protein family, which comprises LMO1, LMO2, LMO3, and LMO4, is involved in cell differentiation and fate during animal development (Matthews et al., 2013). Also, it is reported that LMO proteins are associated with the adhesion plaque and actin microfilament organization (Dawid et al., 1998). Although LMO proteins in the nucleus lack a DNA-binding domain, they collaborate with other transcription factors to form a complex to modulate the transcription of target genes. Wagner et al. (2021) reported that LMO3 promoted the development of human adipose tissue by modulating the transcriptional activity of PPARγ, which is a key adipogenic master switch. Moreover, LMO3 overexpression enhanced human adipose-derived stem cell osteogenesis through PI3K/Akt signaling (Kang and Pei, 2022). Recently, LMO proteins have been emerging as key molecules in a wide variety of human cancers. Specifically, some reported that LMO3 contributes to the progression of human neuroblastoma *via* interacting with helix–loop–helix protein 2 (HEN2) (Aoyama et al., 2005). Moreover, LMO3 directly interacts with LATS1 and suppresses Hippo signaling to promote hepatocellular carcinoma invasion and metastasis (Cheng et al., 2018). But limited results have delineated the clinical implications and molecular functions of LMO3 in PCa. Due to their structural similarity, LMO proteins unsurprisingly share some common biological functions, suggesting that LMO3 could show functions similar to those of other LMO proteins. Gu et al. (2015) found that LMO1 appears to be a coactivator of the androgen receptor (AR) involved in the progression of PCa and could be an undeveloped molecular biomarker of prognosis. LMO2, another LMO protein, is reported to regulate cell fate and control cell growth and differentiation *via* repression of E-cadherin expression in PCa (Ma et al., 2007). Thus, whether LMO3 owns its unique cellular features, such as interacting proteins, gene targets, and prognostic value in PCa, needs to be investigated.

In recent years, increasing evidence has addressed the importance of the tumor microenvironment (TME) in the development and progression of PCa (Sfanos, 2022; Wang et al., 2022). In fact, the PCa microenvironment is thought to have fewer tumor-infiltrating immune cells than immunologically ‘hot’ cancers, such as melanoma, bladder, and lung cancers (Stultz and Fong, 2021). Even so, the infiltration of specific immune cells in PCa has a link with prognosis and response to immunotherapy (Hempel Sullivan et al., 2021; Weiner et al., 2021). These results suggested that the interaction between the tumor cells and TME might be of much importance in PCa. Therefore, there is urgency in precisely indicating the dynamic modulation of the TME.

In this study, we visualized the expression of LMO3 using multiple databases including TIMER, GEPIA2, UALCAN, GEO, and Kaplan–Meier plotter. We then integrated several bioinformatics analyses to explore the correlation between LMO3 and PCa progression and immune infiltration to review its molecular function.

## 2 Methods

### 2.1 LMO3 expression in TIMER, GEPIA2, UALCAN, TCGA, and GEO

In this study, LMO3 expression in pan-cancer was assessed in TIMER (https://cistrome.shinyapps.io/timer/) (Li et al., 2017), GEPIA2 (http://gepia2.cancer-pku.cn/) (Tang et al., 2019), and UALCAN (http://ualcan.path.uab.edu/) (Chandrashekar et al., 2017). GEPIA2 is based on TCGA and GTEx projects, while UALCAN is based on TCGA and MET500 data. We also downloaded and analyzed RNA sequencing data on PCa from TCGA (https://portal.gdc.cancer.gov/) by the “DESeq2” package (Love et al., 2014) in R software (version 3.6.3). To illustrate the expression of LMO3, GSE30994 and GSE70769 were re-analyzed from the GEO database (https://www.ncbi.nlm.nih.gov/geo/) by using the “limma” package (Ritchie et al., 2015).

To further validate the relationship between LMO3 expression and different clinical parameters, we compared their expression profiles regarding age, race, PSA, TNM stage, primary therapy outcomes, residual tumor, and Gleason score by the Kruskal–Wallis test. *p*-values < 0.05 were considered statistically significant.

### 2.2 The correlation between LMO3 and survival

To identify the prognostic value of LMO3 in PCa, we performed a log-rank test and univariate Cox regression for survival analysis with clinical data from TCGA and GSE70769 by using the “survival” package. Moreover, survival maps and Kaplan–Meier survival curves in other cancer types were performed to prove that LMO3 may be a promising prognostic biomarker by GEPIA2 and Kaplan–Meier plotter (http://kmplot.com/analysis/). Specifically, the patients were separated into two groups (high- and low-LMO3 groups) by median expression to analyze the progression-free survival (PFS), disease-free survival (DFS), or overall survival (OS).

To better apprehend the prognostic value of LMO3 in PCa, we divided patients in TCGA database into subgroups based on clinical parameters. In each subgroup, the correlation between LMO3 expression and PFS in patients with PCa was analyzed using the Kaplan–Meier curves. The hazard ratio (HR) with 95% confidence interval and log-rank *p*-values were calculated. *p*-values < 0.05 were considered statistically significant.

### 2.3 Functional enrichment analysis and analysis of the LMO3-interacting network

GO and KEGG analyses were conducted to explore molecular functions of LMO3 in PCa. The potential mechanisms of LMO3 on PCa were investigated by GSEA (Subramanian et al., 2005). All these were performed by using the “clusterProfiler” package (Yu et al., 2012). To analyze LMO3-interacting genes and proteins, we used GeneMANIA (http://www.genemania.org) and STRING (https://string-db.org/) to construct an interaction network of LMO3. Adjusted *p*-values < 0.05 were considered statistically significant.

### 2.4 Correlation analysis between LMO3 expression and the tumor microenvironment

The correlation between LMO3 and immune cell infiltration in PRAD was analyzed in the “Gene” module of TIMER. We also investigated the correlation between LMO3 expression and various immune cells’ gene markers with the “Correlation” module with purity or age-adjusted Spearman’s correlation.

To further illustrate the relationship between LMO3 expression and the TME, a single-sample Gene Set Enrichment Analysis (ssGSEA) algorithm was applied to comprehensively evaluate the immunological characteristics of each sample with the “GSVA” package (Hänzelmann et al., 2013). Moreover, we also calculated the StromalScore, ImmuneScore, and ESTIMATEScore with the “estimate” package (Yoshihara et al., 2013). *p*-values < 0.05 were considered statistically significant.

### 2.5 Drug response of chemotherapy, endocrine therapy, and immunotherapy

To explore the drug sensitivity of chemotherapy, endocrine therapy, and immunotherapy, the clinical responses of two groups stratified based on the expression of LMO3 were predicted and analyzed. The Genomics of Drug Sensitivity in Cancer (GDSC) database was used to predict the response to some chemotherapeutic and endocrine therapy drugs with the “pRRophetic” R package (Yang et al., 2012). To cover more drugs, Cancer Therapeutics Response Portal (CTRP) data, which were prepackaged into the “oncoPredict” R package, were used (Maeser et al., 2021).

As for immunotherapy, the Tumor Immune Dysfunction and Exclusion (TIDE) score was calculated online (http://tide.dfci. harvard.edu/) to evaluate the potential clinical efficacy of immunotherapy (Fu et al., 2020). Subsequently, we used immunophenoscore (IPS) to detect the characteristics of the tumor immune landscape (Charoentong et al., 2017). IPS was used to detect the efficacy of anti-CTLA-4 and anti-PD-1 treatment regimens.

### 2.6 Cell culture, RNA isolation, and qPCR

The human prostate epithelial cell line RWPE-1 and PCa cell lines PC-3, DU145, and VCaP were cultured in 1640 medium (Gibco, CA, United States) supplemented with 10% fetal bovine serum (FBS) and 1% penicillin/streptomycin, incubated with 5% CO_2_ at 37°C. Total RNA was extracted as previously described (Gao et al., 2021). qPCR was applied to measure RNA levels with three independent experiments. Primers for LMO3 (forward, 5′-GAC​ACC​AAG​CCG​AAA​GGT​TG-3′, reverse, 5′-ATG​CCA​GTA​TTT​GTC​CAG​TGC-3′) and β-actin (forward, 5′-AGC​GGG​AAA​TCG​TGC​GTG​AC-3′, reverse, 5-AGG​AAG​GAA​GGC​TGG​AAG​AGT​G-3′) were used for qPCR. *p*-values < 0.05 were regarded as statistically significant.

### 2.6 Western blot

Total proteins were extracted as previously described (Li et al., 2021). Then, 20 μg protein lysate was subjected to sodium dodecyl sulfate–polyacrylamide gel for electrophoresis and then transferred to a polyvinylidene difluoride membrane. After blocking in 5% bovine serum albumin for 1 h, the membranes were incubated overnight at 4°C with primary antibodies against β-actin (ABclonal, AC026, Wuhan, China) and LMO3 (Servicebio, GB113144, Wuhan, China). After hybridization with secondary antibodies (Boster, BA1056, Wuhan, China) at room temperature, the protein bands were detected with ECL substrate (Servicebio, G2014, Wuhan, China). Three independent experiments were performed.

### 2.7 Immunohistochemistry

We obtained patients’ consent and approval from the Institutional Research Ethics Committee, then collected PCa tissues, and matched adjacent normal tissues from Tongji Hospital, Tongji Medical College, Huazhong University of Science and Technology. Then, immunohistochemistry (IHC) staining was performed using the VECTASTAIN EliteABC kit (Vector Laboratories, Burlingame, CA, United States), and its procedures were presented, as previously described (Gao et al., 2021). Briefly, the sections of three pairs of prostate cancer and adjacent normal prostate tissues were used, and a pathologist ensured the typicality of the selected tissues.

## 3 Results

### 3.1 LMO3 expression is decreased in PCa

**FIGURE 1:**
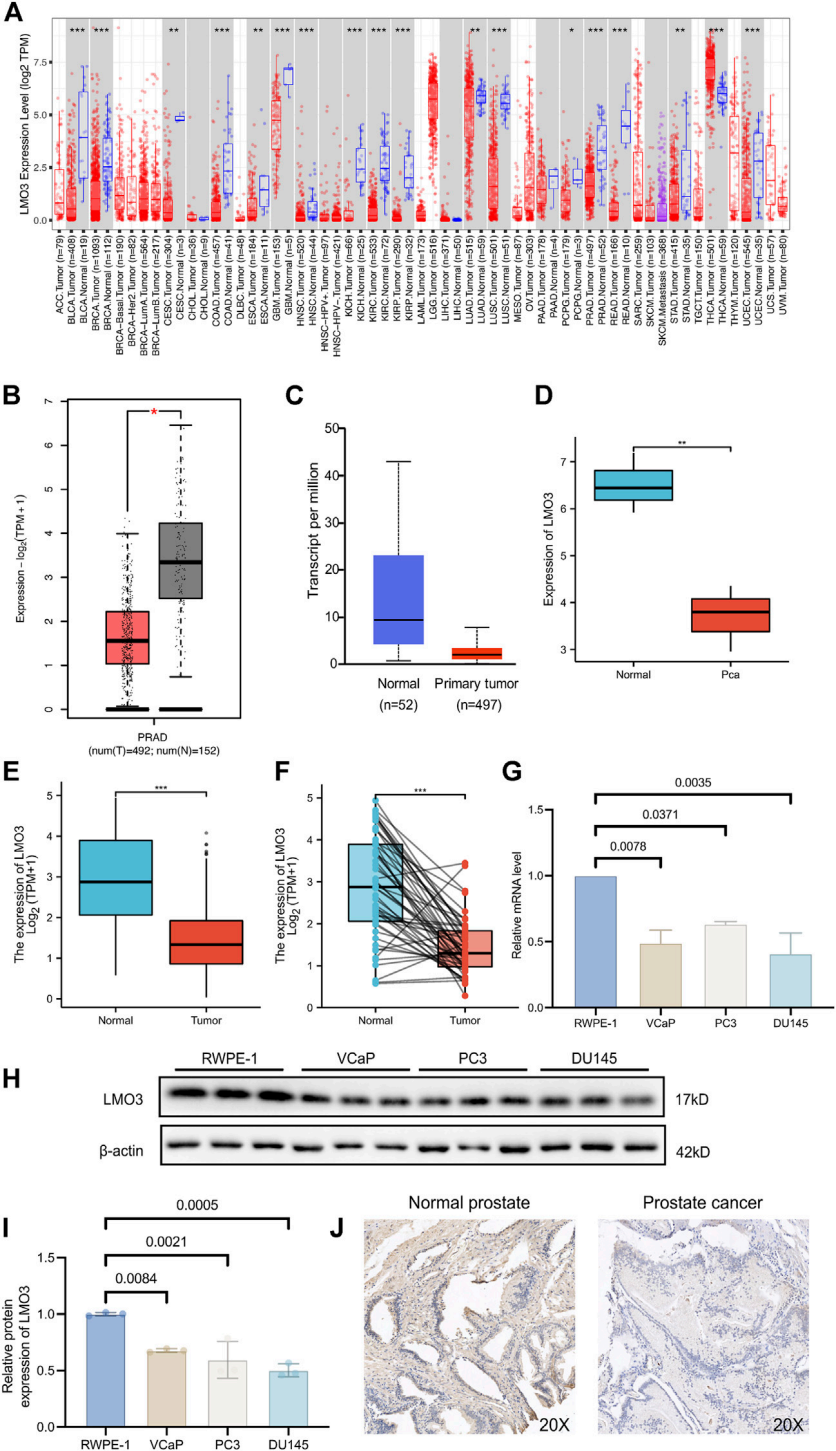
Expression of LMO3 in prostate cancer. **(A)** LMO3 expression in different types of cancer was investigated with the TIMER database. **(B)** Decreased expression of LMO3 in prostate cancer compared to normal tissues in the GEPIA database. **(C)** LMO3 expression in prostate cancer was examined by using the UALCAN database. **(D)** LMO3 expression in prostate cancer was examined by GSE30994. **(E)** Analysis of LMO3 expression in prostate cancer and adjacent normal tissues in TCGA database. **(F)** TCGA database and statistical analyses of LMO3 expression in 52 pairs of PRAD tissues and adjacent normal tissues. **(G)** LMO3 expression in four different cell lines was examined by qPCR. The mean ± s.d. is shown. **(H)** Representatve immunoblot and **(I)** semi-quantification of LMO3 protein expression in four different cell lines **(J)** Immunohistochemical staining of LMO3 was performed in prostate cancer and normal prostate tissues. Representative images are shown. Statistical significance was determined using one-way ANOVA with the *post hoc* Tukey test. *p < 0.05, **p < 0.01, and ***p < 0.001.

The mRNA expression of LMO3 in pan-cancer was first analyzed by the TIMER database. Lower expression of LMO3 was observed in various cancer types, including prostate adenocarcinoma (PRAD), compared with normal tissues (Figure 1A). The results from GEPIA2 and UALCAN showed that the expression of LMO3 was lower in PCa than in normal prostate tissues (Figures 1B,C). A similar result was observed in PCa tissues from GSE30994 (Figure 1D). In addition, re-analysis with data directly obtained from TCGA showed that LMO3 expression was significantly reduced in PCa tissues (Figure 1E). Furthermore, 52 paired samples in PRAD displayed a marked decrease in LMO3 expression in PCa (Figure 1F). In conclusion, these results demonstrate that LMO3 expression is downregulated in PCa and denote that LMO3 may play an essential role in PCa progression. Furthermore, we found that LMO3 expression was significantly downregulated in PCa cell lines compared with nonmalignant ones (Figures 1G–I). The protein expression of LMO3 was further investigated by IHC, and we found that LMO3 was obviously decreased in prostate cancer tissues compared with normal prostate tissues (Figure 1J). To investigate whether other LMO genes are changed in Pan-cancer, the mRNA expression levels of LMO1, LMO2, and LMO3 were significantly observed. Moreover, similar to LMO3, the three LMO genes were downregulated in PRAD (Supplementary Figure S1).

### 3.2 LMO3 expression and clinical characteristics of PCa patients

**FIGURE 2:**
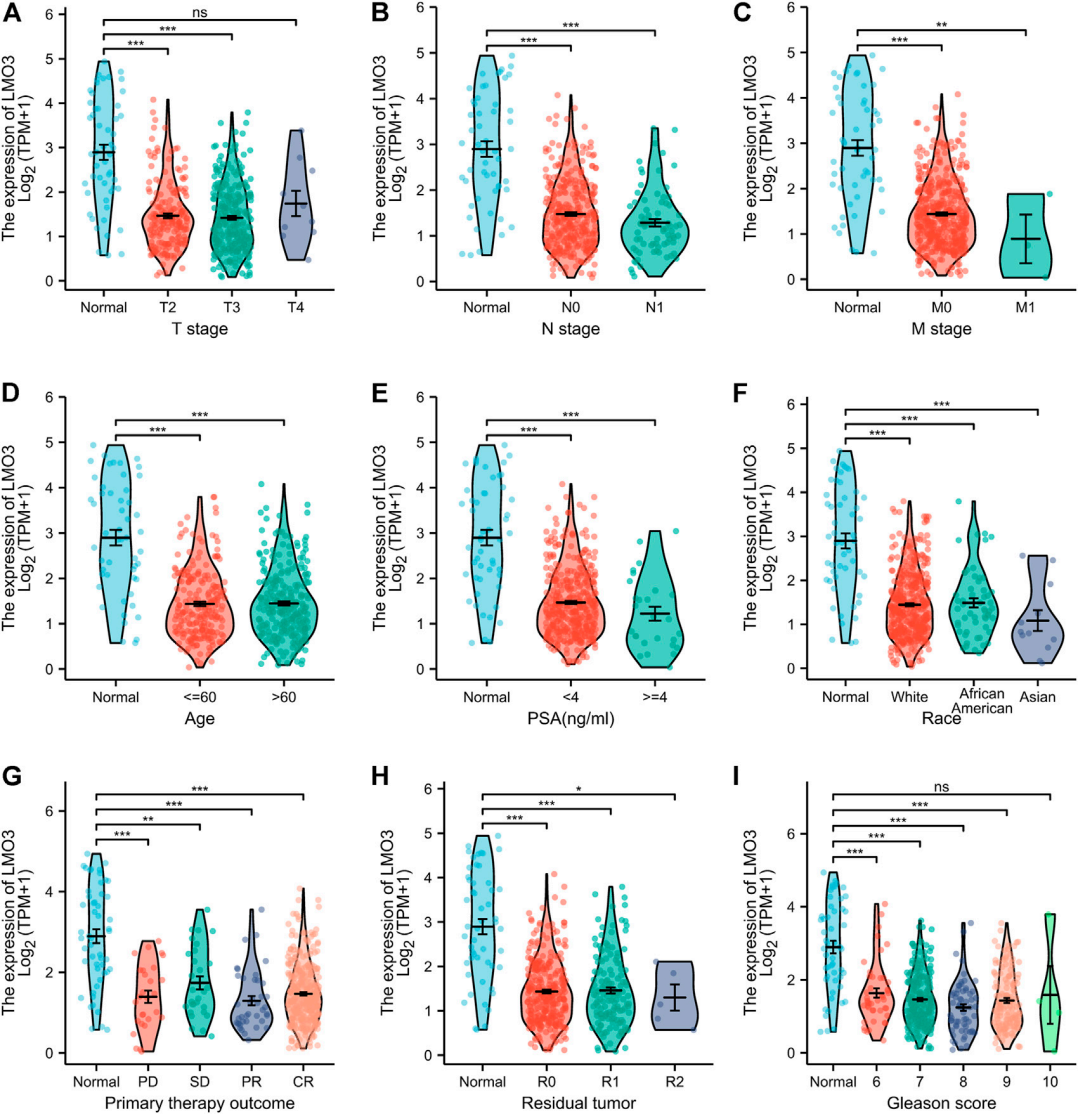
Box plots evaluating LMO3 expression among different groups of patients based on clinical parameters. Analysis is shown for tumor stage **(A)**, cancer stage **(B)**, metastasis **(C)**, age **(D)**, PSA **(E)**, Race **(F)**, primary therapy outcomes **(G)**, residual tumor **(H)** Representatve immunoblot and **(I)** semi-quantification of LMO3 protein expression in four different cell lines. **(J)** Immunohistochemical staining of LMO3 was performed in prostate cancer and normal prostate tissues. Representative images are shown.

We then investigated LMO3 expression on the basis of clinical characteristics. Regarding tumor stage, decreased LMO3 expression was observed in PCa patients in stages 2, 3, and 4 (Figure 2A). LMO3 expression was lower, regardless of whether there is lymph node invasion and metastasis or not (Figures 2B,C). In terms of age, the LMO3 level was significantly reduced in the PCa tissues from different groups (Figure 2D). According to PSA, LMO3 expression was significantly downregulated in PCa samples from both <4 and >=4 ng/ml compared to the corresponding normal controls (Figure 2E). In addition, LMO3 expression was dramatically decreased in Asian PCa patients (Figure 2F). According to primary therapy outcomes and residual tumor, LMO3 expression was reduced in PRAD patients (Figures 2G,H). Moreover, downregulation of LMO3 expression was observed in PRAD cancer patients with Gleason scores of 6, 7, 8, and 9 compared to normal controls (Figure 2I). These findings imply that LMO3 expression is inseparably correlated with tumor progression.

### 3.3 Decreased LMO3 expression correlates with unfavorable prognosis

**FIGURE 3:**
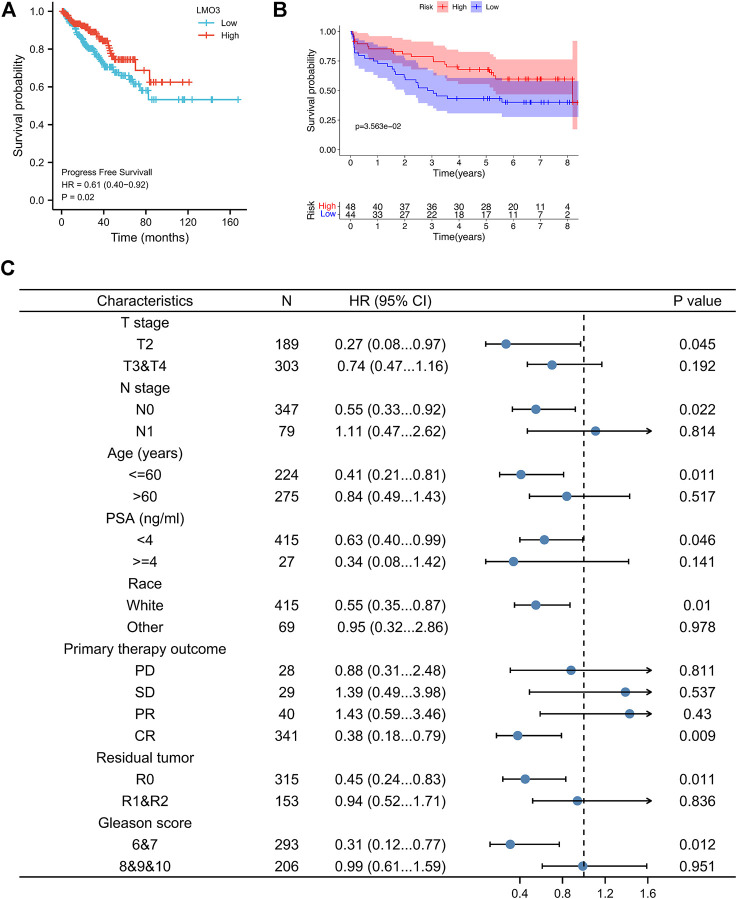
Survival curve evaluating the prognostic value of LMO3. **(A)** Survival curves are shown for PFS. **(B)** Survival curves using the GSE70769 cohort are shown for DFS. **(C)** Forest plot showing the correlation between LMO3 expression and clinical parameters in PRAD patients.

Since the LMO3 expression is closely related to PCa progression, we examined the prognostic value of LMO3. Lower LMO3 expression exhibited unfavorable progression-free survival (PFS) in PCa (Figure 3A). Moreover, decreased expression of LMO3 was significantly associated with unfavorable disease-free survival (DFS) in the GSE70769 cohort (Figure 3B). These findings indicate that LMO3 is considerably related to the prognosis of PCa. To further prove that LMO3 may be a prospective prognostic biomarker, we performed survival maps and Kaplan–Meier survival curves in other cancer types. As the figures demonstrated, LMO3 was significantly associated with the prognosis of kidney renal papillary cell carcinoma and sarcoma based on DFS (Supplementary Figure S2). Regarding overall survival (OS), LMO3 was related to the prognosis of kidney renal clear cell carcinoma, liver hepatocellular carcinoma, lung adenocarcinoma, and uterine corpus endometrial carcinoma (Supplementary Figure S3).

To better apprehend the prognostic value of LMO3 in PCa, we evaluated the relationship between LMO3 mRNA expression and clinical parameters (Figure 3C). Regarding TNM grade, low LMO3 expression was correlated with unfavorable PFS in T2 and N0 PCa patients. For PCa patients under 60 years of age, LMO3 downregulation was associated with unfavorable PFS. The correlation between LMO3 expression and poor PFS was significantly observed in PCa patients with PSA <4 ng/ml. Moreover, we found a significant association between LMO3 expression and poor PFS in white patients. Low LMO3 expression was correlated with unfavorable PFS in patients with complete response (CR) and R0 (no residual tumor). In addition, downregulated LMO3 corresponded with unfavorable PFS in patients with Gleason scores of 6 and 7. These findings implicate that LMO3 expression exhibits a good prognostic value in PCa.

### 3.4 LMO3-interacting gene and functional enrichment analysis

**FIGURE 4:**
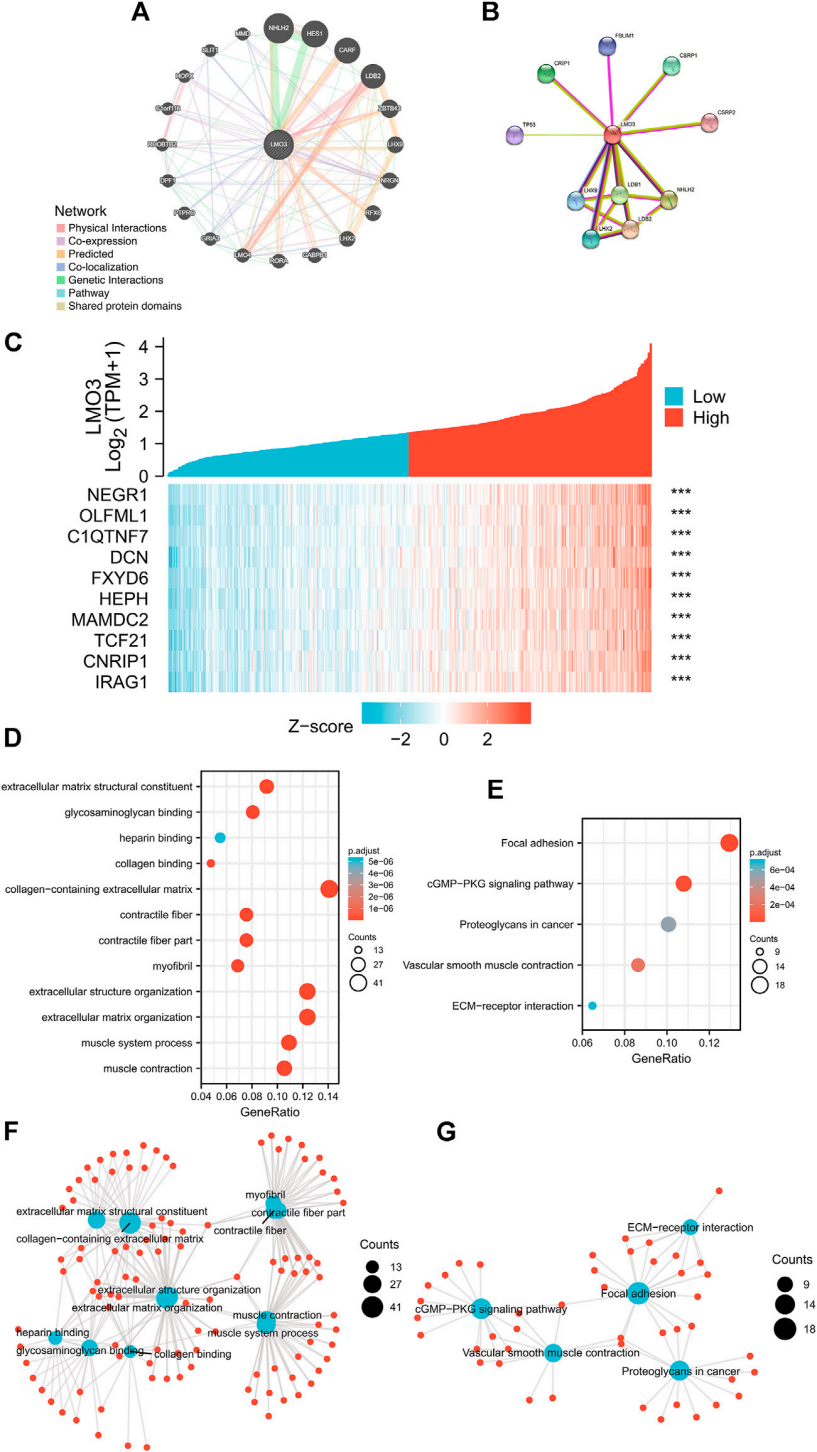
**(A)** Gene–gene interaction network of LMO3 was constructed using GeneMANIA. **(B)** PPI network of LMO3 was generated using STRING. **(C)** Top 10 genes correlated with LMO3 in PRAD. **(D)** and **(F)** show top 20 enrichment terms in GO. **(E)** and **(G)** show top 20 KEGG enrichment pathways. *p < 0.05, **p < 0.01, and ***p < 0.001.

We generated the gene–gene interaction and protein–protein interaction (PPI) network for LMO3 by GeneMANIA and STRING. The results demonstrated that NHLH2, HES1, LHX9, and CARF most frequently interact with LMO3 (Figure 4A). The PPI network of LMO3 showed 11 nodes, including NHLH2, LHX9, and LDB2 (Figure 4B). To further confirm whether LMO3 influences these genes in PCa, we compared the expression between normal and tumor samples (Supplementary Figure S4). The results showed that many of them were altered, suggesting that LMO3 might mediate their expression and function in PCa.

Based on data from TCGA, the top 10 genes that are most relevant to LMO3 in PRAD are shown in Figure 4C. To depict LMO3-involved pathways and molecular functions, 300 positively correlated genes were used for functional enrichment analysis. (Figures 4D–G).

### 3.5 GSEA marked LMO3-involved pathways

**FIGURE 5:**
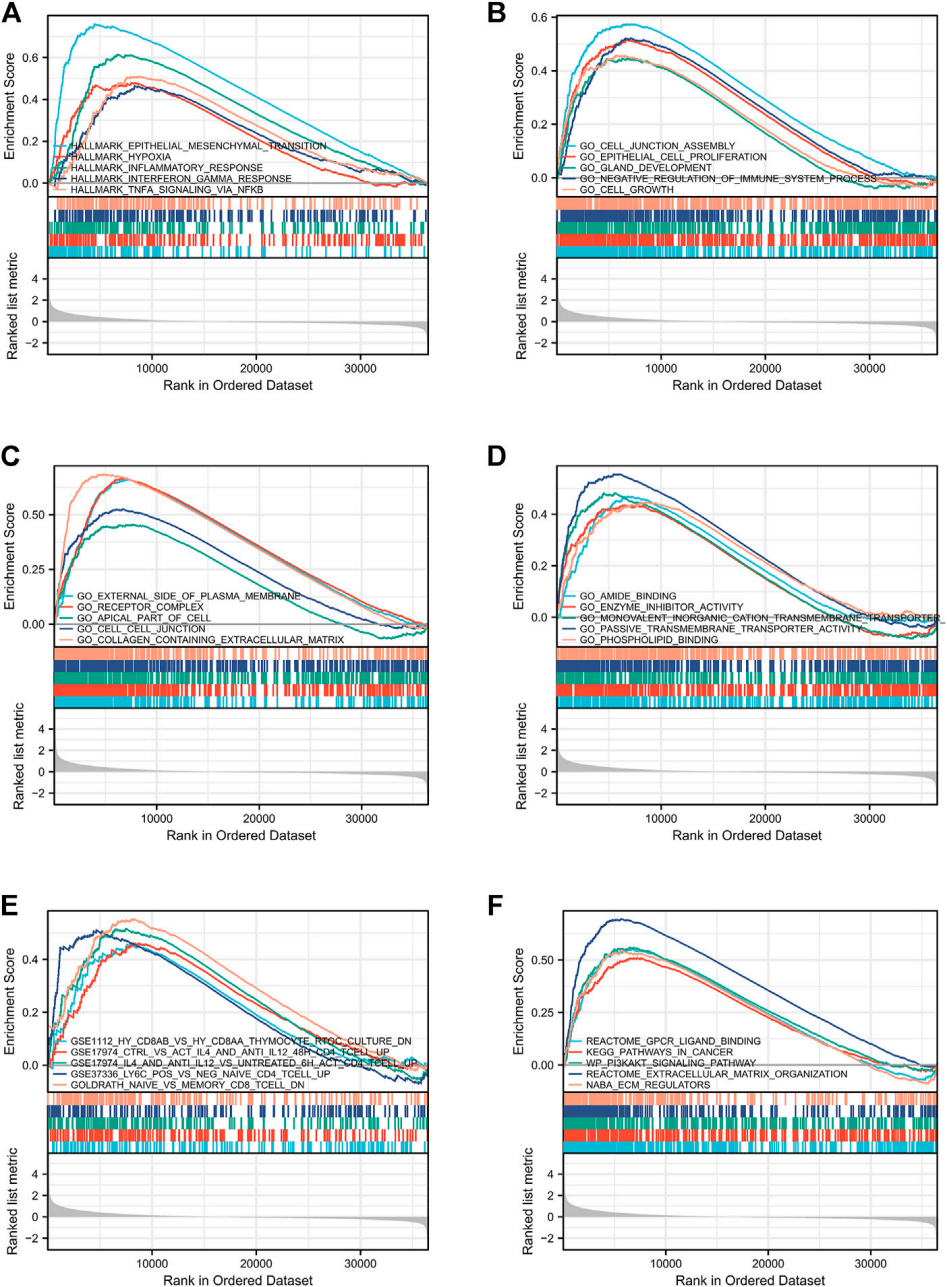
Enrichment plots from GSEA. The pathways associated with LMO3 expression based on hallmark gene sets **(A)**, GO terms **(B–D)**, C7 collection **(E),** and KEGG terms **(F)**.

To check out the molecular mechanisms of LMO3 in PCa, we conducted a GSEA analysis. Epithelial–mesenchymal transition (EMT), hypoxia, inflammatory response, interferon-gamma response, and TNFα signaling were the top five LMO3-involved pathways in hallmark gene sets defined by MSigDB (Figure 5A). Among the GO terms, negative regulation of the immune system process, cell–cell junction, and enzyme inhibitor activity were enriched (Figures 5B–D). For the C7 collection, the immunologic gene sets and multiple immune functional gene sets were enriched (Figure 5E). Among the KEGG terms, GSEA-revealed pathways in cancer, extracellular matrix organization, and ECM regulators were enriched (Figure 5F). To further investigate the function of LMO3 in PCa, we stratify PRAD patients into two groups based on the expression of LMO3 to dig out what pathways are getting differentially enriched. Similarly, GO and KEGG analyses showed that the ECM–receptor interaction, inflammatory mediator regulation of TRP channels, extracellular structure organization, extracellular matrix structural constituent, and collagen-containing extracellular matrix were enriched (Supplementary Figure S5). These findings firmly implicate that LMO3 regulates the extracellular matrix and immune response in PCa.

### 3.6 Correlation analysis between LMO3 expression and the TME

**FIGURE 6:**
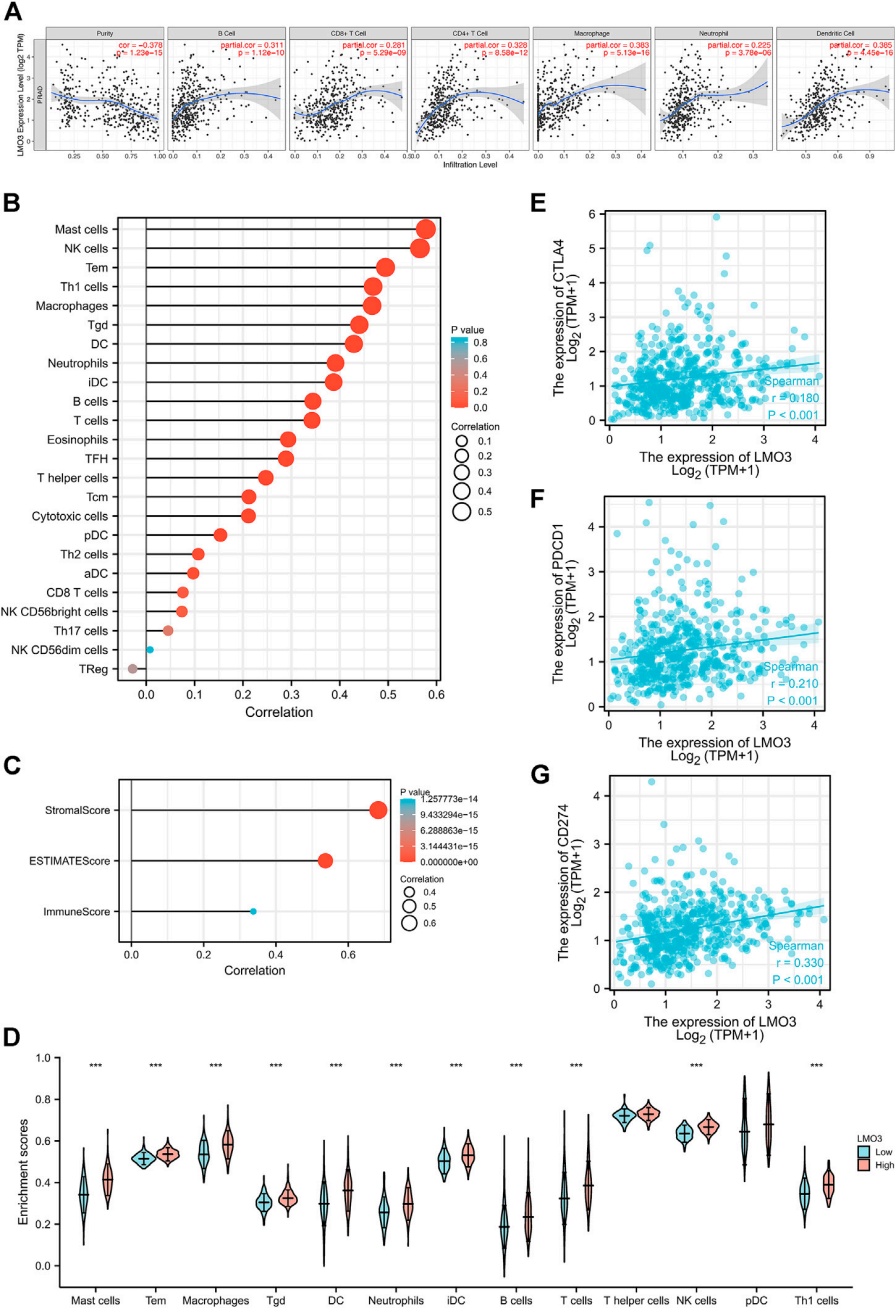
Correlation of LMO3 expression with the immune infiltration level. **(A)** LMO3 is positively correlated with the infiltration of different immune cells using the TIMER database. LMO3 expression has a significant correlation with the infiltration of immune cells in prostate cancer by ssGSEA **(B)** and ESTIMATE **(C)**. **(D)** Subgroup analyses demonstrated that 11 kinds of immune cells were positively correlated with the expression of LMO3. **(E–G)** Scatterplots of the correlations between LMO3 expression and PD-1, PD-L1, and CTLA-4 in PRAD.

We explored the correlation between LMO3 expression and immune cell infiltration in TIMER. The results demonstrated that LMO3 expression is positively correlated with the infiltration of six types of immune cells in PRAD (Figure 6A). To further evaluate the influence of LMO3 on the TME, we assessed the correlation between LMO3 and immune infiltration by “ssGSEA” and “estimate.” Notably, LMO3 was positively related to the infiltration levels of mast cells, NK cells, Tem, Th1 cells, and macrophages (Figure 6B). Moreover, LMO3 was positively correlated with the stromal score, estimate score, and immune score (Figure 6C). Subgroup analyses demonstrated that 11 kinds of immune cells were positively correlated with the expression of LMO3 (Figure 6D).

In addition, we estimated the relationship between LMO3 expression and T-cell checkpoints, including PD-1, PD-L1, and CTLA-4. LMO3 expression was notably correlated with these markers in PRAD (Figures 6E–G). These results validate that LMO3 expression is significantly correlated with immune infiltration and imply that LMO3 plays an essential role in immune escape in the TME of PCa.

### 3.7 LMO3 expression and immune cell markers

To strengthen our comprehension of the LMO3 interaction with the immune response, the correlation between LMO3 and various immune markers in PRAD was assessed in TIMER. We listed the genes characterizing immune cells, including mast cells, natural killer (NK) cells, macrophages, dendritic cells (DC), neutrophils, B cells, T cells, and monocytes in Table1. Tumor purity and age are two important factors influencing the analysis of immune infiltration in tumor samples. After adjusting for tumor age or purity, LMO3 expression was markedly correlated with most markers of immune cells in PRAD (Table 1).

**TABLE 1:** Correlation analysis between LMO3 and gene markers of immune cells in TIMER.

Description	Gene marker	None		Purity		Age	
		Cor	p	Cor	P	Cor	P
Mast cells	KIT	0.546	***	0.453	***	0.548	***
	ENPP3	−0.132	**	−0.114	*	−0.13	**
Nk cells	NCAM1	0.795	***	0.756	***	0.785	***
	FCGR3A	0.287	***	0.216	***	0.273	***
	KLRD1	0.315	***	0.163	***	0.310	***
Macrophages	CD14	0.364	***	0.271	***	0.354	***
	CD68	0.290	***	0.187	***	0.278	***
	CSF1R	0.477	***	0.370	***	0.469	***
M1	IRF5	0.141	**	0.114	*	0.128	**
	PTGS2	0.244	***	0.149	**	0.239	***
	NOS2	0.103	*	0.001	0.981	0.100	*
M2	CD163	0.336	***	0.237	***	0.327	***
	VSIG4	0.384	***	0.278	***	0.374	***
	MS4A4A	0.372	***	0.268	***	0.365	***
DC	HLA-DPB1	0.380	***	0.256	***	0.368	***
	HLA-DQB1	0.255	***	0.155	**	0.249	***
	HLA-DRA	0.339	***	0.193	***	0.327	***
	HLA-DPA1	0.415	***	0.280	***	0.404	***
	CD1C	0.328	***	0.178	***	0.322	***
	NRP1	0.076	0.091	0.054	0.268	0.064	0.162
	ITGAX	0.245	***	0.139	**	0.233	***
Neutrophils	FCGR3B	0.277	***	0.186	***	0.273	***
	CEACAM8	0.008	0.855	-0.018	0.714	0.009	0.837
	ITGAM	0.439	***	0.330	***	0.429	***
	CCR7	0.276	***	0.124	*	0.274	***
B cells	CD19	0.258	***	0.170	***	0.264	***
	CD79A	0.280	***	0.187	***	0.276	***
T cells (general)	CD3D	0.266	***	0.108	*	0.259	***
	CD3E	0.352	***	0.205	***	0.346	***
	CD2	0.310	***	0.160	**	0.302	***
CD8^+^ T cells	CD8A	0.308	***	0.148	**	0.301	***
	CD8B	0.118	**	0.025	0.607	0.104	*
Monocytes	CD86	0.343	***	0.226	***	0.334	***
	CSF1R	0.477	***	0.370	***	0.469	***

*p < 0.05, **p < 0.01, and ***p < 0.001.

We also validated the connection between LMO3 and different functional T cells, including Th1, Th1-like, Th2, Treg, effector T cells, naïve T cells, and exhausted T cells (Table 2). These results in TIMER showed that the LMO3 expression level was significantly associated with 19 or 22 of 22 T-cell markers after respectively adjusting for tumor purity or age (Table 2).

**TABLE 2:** Correlation analysis between LMO3 and gene markers of different types of T cells in TIMER.

Description	Gene marker	None		Purity		Age	
		Cor	*p*	Cor	*p*	Cor	*p*
Th1	TBX21	0.258	***	0.148	**	0.245	***
	STAT4	0.289	***	0.149	**	0.281	***
	STAT1	0.149	***	0.058	0.237	0.143	**
	TNF	0.181	***	0.060	0.225	0.173	***
	IFNG	0.153	***	0.063	0.197	0.146	**
Th1-like	HAVCR2	0.322	***	0.200	***	0.311	***
	CXCR3	0.253	***	0.131	**	0.242	***
	BHLHE40	0.213	***	0.155	**	0.208	***
	CD4	0.381	***	0.257	***	0.372	***
Th2	STAT6	0.304	***	0.233	***	0.299	***
	STAT5A	0.488	***	0.388	***	0.479	***
Treg	FOXP3	0.190	***	0.121	*	0.186	***
	CCR8	0.182	***	0.108	*	0.178	***
	TGFB1	0.455	***	0.385	***	0.445	***
Effector T cells	CX3CR1	0.371	***	0.284	***	0.362	***
	FGFBP2	0.230	***	0.149	**	0.225	***
	FCGR3A	0.287	***	0.216	***	0.273	***
Naïve T cells	CCR7	0.276	***	0.124	*	0.275	***
	SELL	0.368	***	0.257	***	0.362	***
Exhausted T cells	LAG3	0.278	***	0.183	***	0.273	***
	CXCL13	0.374	***	0.294	***	0.378	***
	LAYN	0.606	***	0.541	***	0.601	***

*p* < 0.05, **p < 0.01, and ***p < 0.001.

### 3.8 Effect of LMO3 on drug sensitivity

**FIGURE 7:**
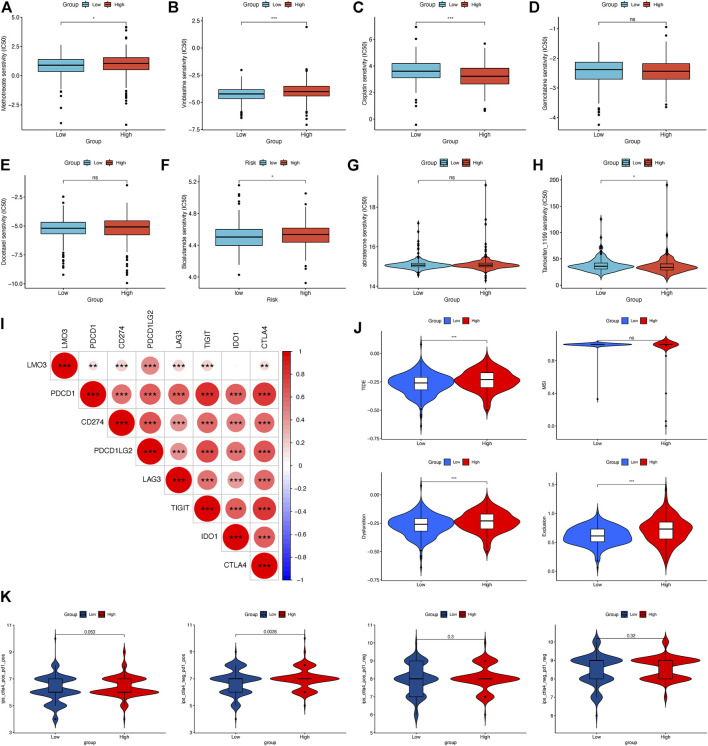
Relationship between LMO3 expression and drug sensitivity. **(A–H)** IC50 of LMO3 expression-defined subgroups to drugs, including **(A)** methotrexate, **(B)** vinblastine, **(C)** cisplatin, **(D)** gemcitabine, **(E)** docetaxel, **(F)** bicalutamide, **(G)** abiraterone, and **(H)** tamoxifen. **(I)** Correlation between LMO3 and immune checkpoint expression. **(J)** TIDE, MSI, and T-cell exclusion and dysfunction scores in different subgroups. **(K)** Differential analysis for different subgroups in immunophenoscore (IPS) with CTLA4 (+)/PD1 (+). *p < 0.05, **p < 0.01, and ***p < 0.001.

We evaluated the efficacy of chemotherapy and endocrine therapy in different subgroups by IC50 values (Figures 7A–H). These results showed that the IC50 value of methotrexate and vinblastine was significantly higher in the high-expression group. Cisplatin was more suitable for low-expression patients. Moreover, the efficacy of gemcitabine and docetaxel was comparable between the two groups (Figures 7A–E). As endocrine therapy is currently the main treatment for PCa, we chose bicalutamide, abiraterone, and tamoxifen to predict the drug response of endocrine therapy. As shown in Figures 7F–H, the low-expression group was likely to benefit from bicalutamide, while less from tamoxifen, and got a similar response to abiraterone. To further dig out the relationship between the LMO3 expression and response to immunotherapy, we calculated the relevance between LMO3 and more checkpoints. As shown in Figure 7I, LMO3 had significantly positive relevance with seven immune checkpoints (PDCD1, CD274, PDCD1LG2, LAG3, TIGIT, IDO1, and CTLA4). Consequently, we speculated that the high-expression group tends to respond effectively to immunotherapy. So we then used TIDE and IPS to assess the potential clinical efficacy of immunotherapy in different subgroups. Higher scores of TIDE, MSI, dysfunction, and exclusion represented a higher potential for immune evasion. The results of TIDE demonstrated that the LMO3 low-expression group had a lower score, implying that the LMO3 low-expression patients could benefit more from immunotherapy (Figure 7J). In addition, IPS results showed that LMO3 high-expression patients were more likely to respond effectively to anti-PD-1 immunotherapy (Figure 7K). The prediction method may account for the subtle difference between TIDE and IPS. Therefore, more functional experiments and clinical data are urgent.

## 4 Discussion

PCa is one of the most commonly diagnosed malignancies worldwide (Løvf et al., 2019; Sung et al., 2021). This rise in prevalence has been compounded by population growth and aging (Tseng, 2011). Prostate-specific antigen (PSA), TNM stage, and Gleason score are widely used as prognostic markers of PCa in a clinic. However, none of them alone or in combination can meet the needs of clinical prognostic assessment of PCa. In this study, we attempted to identify LMO3 as a perspective prognostic maker in PRAD. These results showed that the LMO3 expression was significantly decreased and associated with age, clinical stage, histological grade, and metastasis in PCa patients. Furthermore, low LMO3 expression exhibited a markedly unfavorable prognosis. Overall, a series of bioinformatics analyses confirmed that LMO3 may have a chance to be an independent prognostic biomarker of PCa and promote the precision oncology of PCa.

In recent years, research about molecular typing of cancer has been widely carried out in many kinds of tumors. It has made tumor classification change from traditional morphology to molecular typing based on molecular characteristics (Blattner et al., 2017; Moreira et al., 2018; Reimers et al., 2020). Molecular typing of tumors plays an important role in guiding clinical decision-making, in which the key step is to find more effective molecular markers related to tumor prognosis. Human LMO3 is highly expressed in the brain. In addition to the brain, LMO3 is also detected in other tissues and organs, such as the colon, bladder, lungs, and prostate. Several studies have reported that LMO3 is involved in neuroblastoma (Aoyama et al., 2005) and hepatocellular carcinoma (HCC) (Cheng et al., 2018). For example, LMO3 expression is significantly upregulated in HCC. It interacts with LATS1 to suppress the Hippo pathway, acting as an oncogene to promote HCC cell proliferation, invasion, and metastasis (Cheng et al., 2018). In this study, we found that LMO3 was abnormally expressed and associated with the prognosis of many cancers, suggesting that it may be involved in tumorigenesis and development. In addition, we also verified that LMO3 was downregulated in PCa tissues and cell lines. These results imply that LMO3 may function as a promising marker and a tumor suppressor gene in PCa, and functional LMO3 is decomposed to promote PCa proliferation. This is obviously different from the cancer-promoting function of LMO3 in other types of tumors (Aoyama et al., 2005; Cheng et al., 2018). Therefore, the precise molecular mechanisms of LMO3 in PCa still need to be further explored.

In the process of tumorigenesis and development, the TME interacts with tumor cells to mediate the immune tolerance of the tumor, thus affecting the clinical effect of immunotherapy (Strasner and Karin, 2015; Kwon et al., 2021). Removing the immunosuppression of the TME is beneficial to the recovery and reconstruction of the normal anti-tumor immune defense ability of the human body, thus enhancing the comprehensive efficacy of various tumor treatment methods, including immunotherapy (Wang et al., 2022). It is of much importance to identify the prospective therapeutic targets resulting in remodeling of the TME and transition of the TME from being tumor-friendly to tumor-suppressing (Bi et al., 2020). So far, the association between LMO3 and immune cell infiltration in PCa has not been explored. Here, we first found that LMO3 expression is correlated with the immune components in the TME. In other words, the proportion of immune components in the TME is significantly correlated with the progression of PCa (Wu et al., 2020). In particular, high M1 macrophages and neutrophils are associated with patients’ prognosis, suggesting that these two immune cells might be potential targets in PCa (Wu et al., 2020). These results imply that LMO3 could be a potential immunotherapy target in PCa. However, the exact role of LMO3 in the TME still requires in-depth investigation.

This study enhances our understanding of the connection between LMO3 and PCa; however, a few constraints still exist. First, in spite of the fact that we observed that LMO3 was rarely expressed in PCa cell lines, the molecular mechanisms of LMO3 in tumor progression, metastasis, and immune infiltration should be investigated in future studies. In addition, although LMO3 was abnormally expressed and associated with the prognosis of many cancers, we need to answer whether the abnormal effect of LMO3 on tumorigenesis is direct or indirect. When LMO3 gets downregulated in PCa, we indeed should confirm its tumor-friendly or tumor-suppressing role with more functional experiments in the near future. Furthermore, these bioinformatics analyses mainly relied on LMO3’s mRNA levels. Additional analysis in view of protein levels might aggravate the determination of additional convincing.

Overall, these findings imply that LMO3 regulates immune cell infiltration and could function as a prospective biomarker for PCa. Therefore, the present study may advance our comprehension of not only the role of LMO3 on the development and progression of PCa but also its clinical applications in predicting PCa prognosis and guiding suitable immunotherapy.

## Data Availability

The data presented in the study are deposited in “GitHub” repository (https://github.com/wcxutj/LMO3). Publicly available datasets were analyzed in this study, these data can be found at: https://cancergenome.nih.gov/ and https://www.ncbi.nlm.nih.gov/geo/ (accession number:GSE30994 and GSE70769).
