# Supplementary material for: LMO3 downregulation in PCa: A prospective biomarker associated with immune infiltration
Source: Front Genet. 2022 Sep 19;13:945151. doi: 10.3389/fgene.2022.945151 (PMC9527341; doi:10.3389/fgene.2022.945151)
Supplement: Supplementary file 1 [file DataSheet2.PDF]

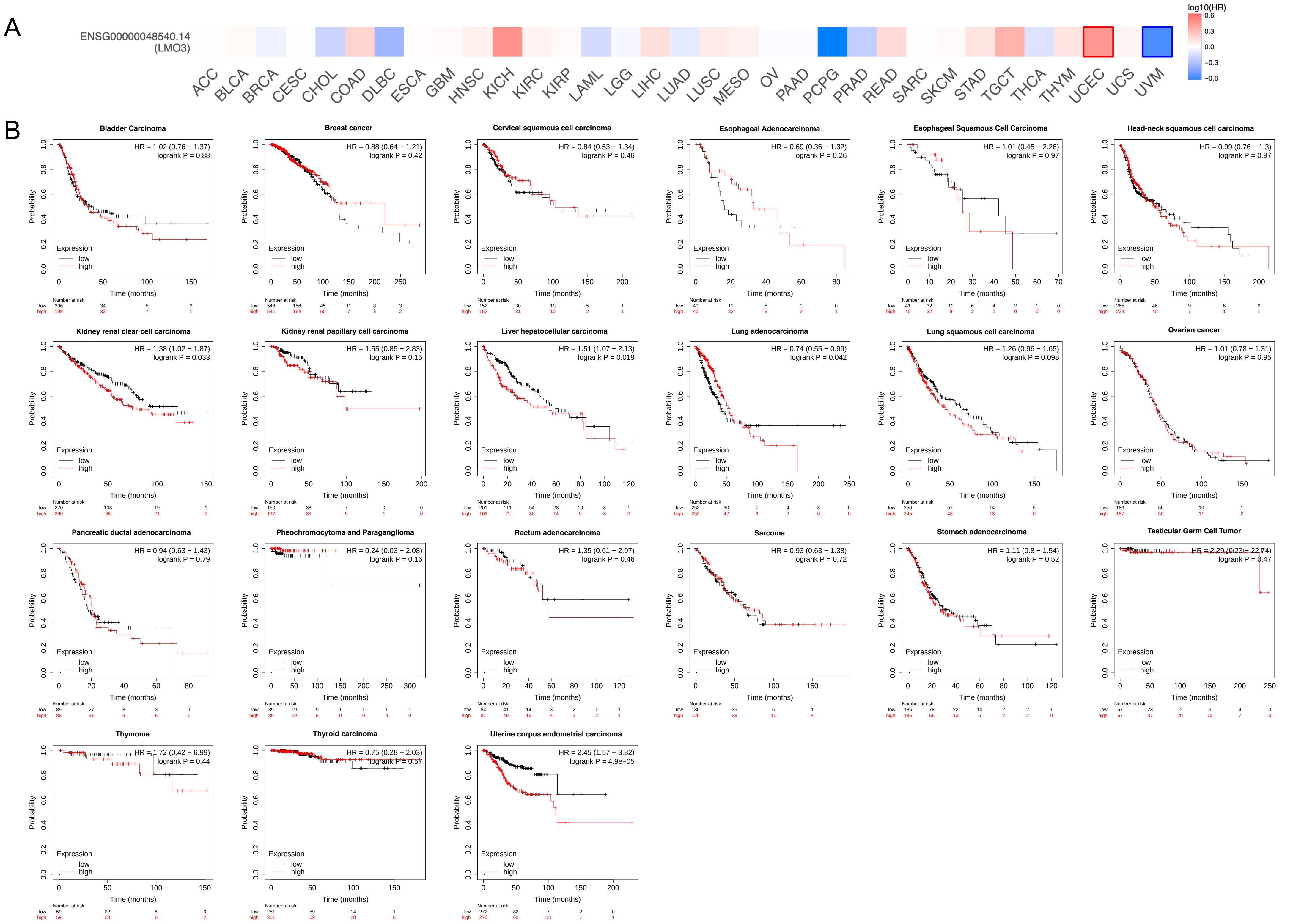

Supplementary figure 2. (A) Survival map in pan-cancer for OS. (B) Survival curve evaluating the prognostic value of LMO3 in pan-cancer for OS.
