# Supplementary material for: LMO3 downregulation in PCa: A prospective biomarker associated with immune infiltration
Source: Front Genet. 2022 Sep 19;13:945151. doi: 10.3389/fgene.2022.945151 (PMC9527341; doi:10.3389/fgene.2022.945151)
Supplement: Supplementary file 2 [file DataSheet4.PDF]

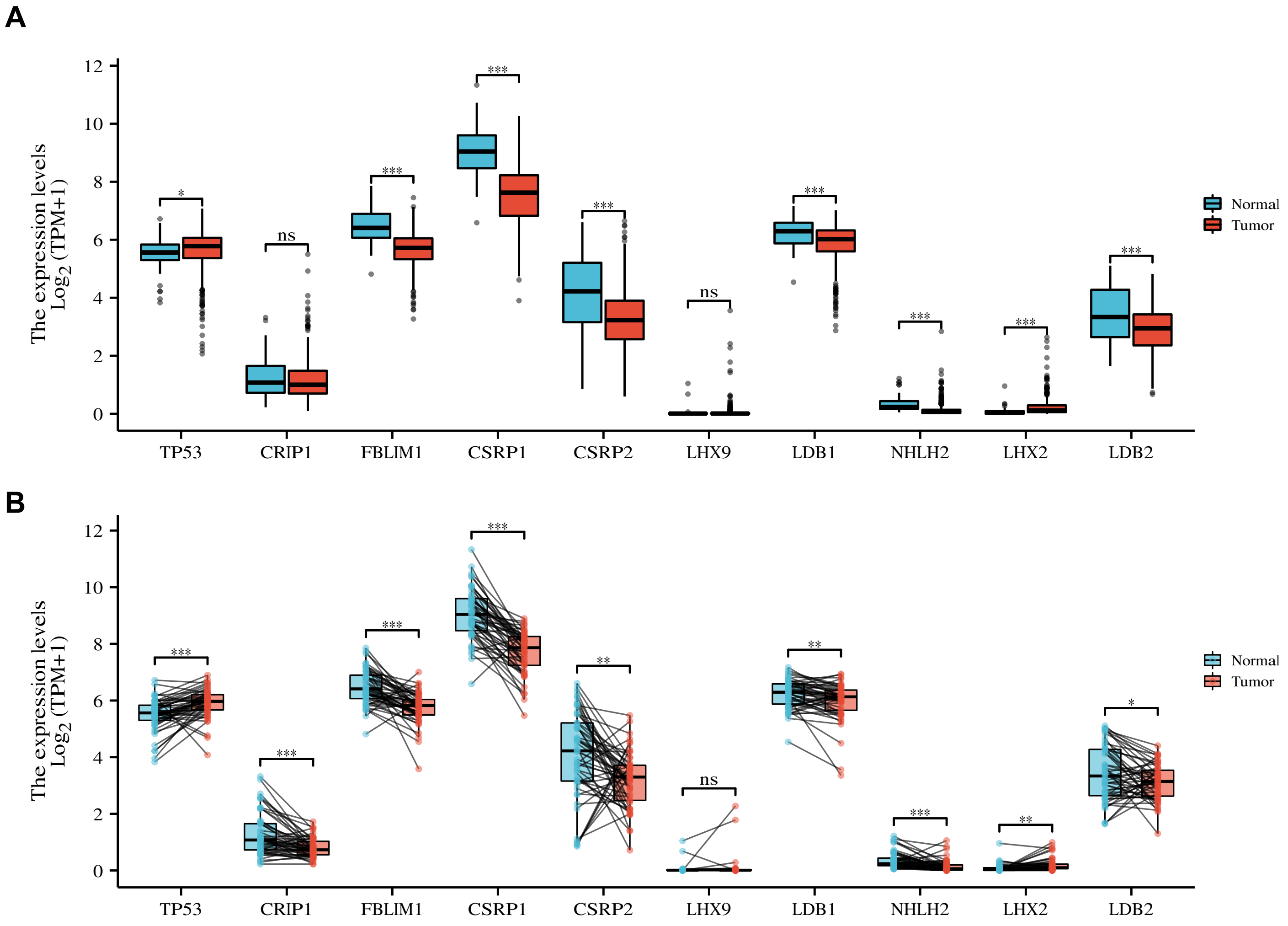

Supplementary figure 4. The expression of LMO3-interacting genes in unpaired (A) and paired (B) in PRAD.
