# Supplementary figures and images for: LMO3 downregulation in PCa: A prospective biomarker associated with immune infiltration
Source: Front Genet. 2022 Sep 19;13:945151. doi: 10.3389/fgene.2022.945151 (PMC9527341; doi:10.3389/fgene.2022.945151)

**A**

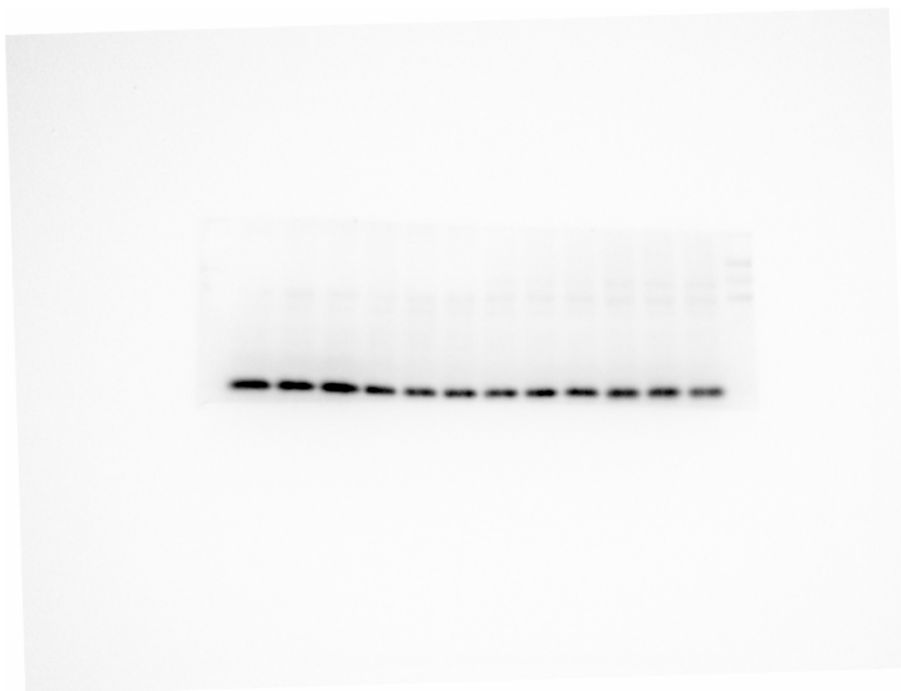

**B**

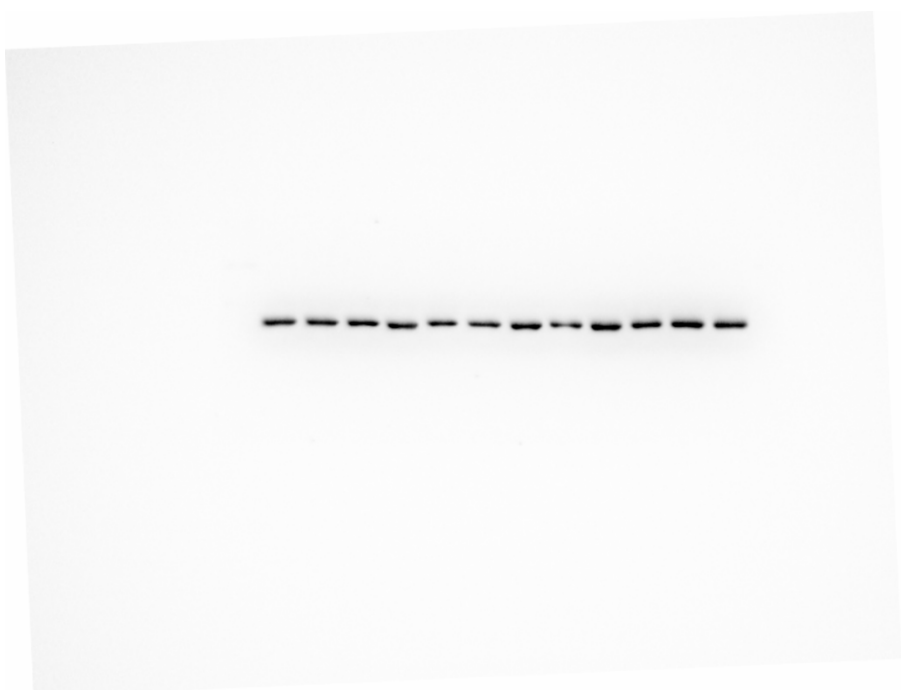

**Supplementary figure x.** Original blotting of LMO3 (A) and actin (B).

Supplement: Supplementary file 3 [file DataSheet6.PDF]

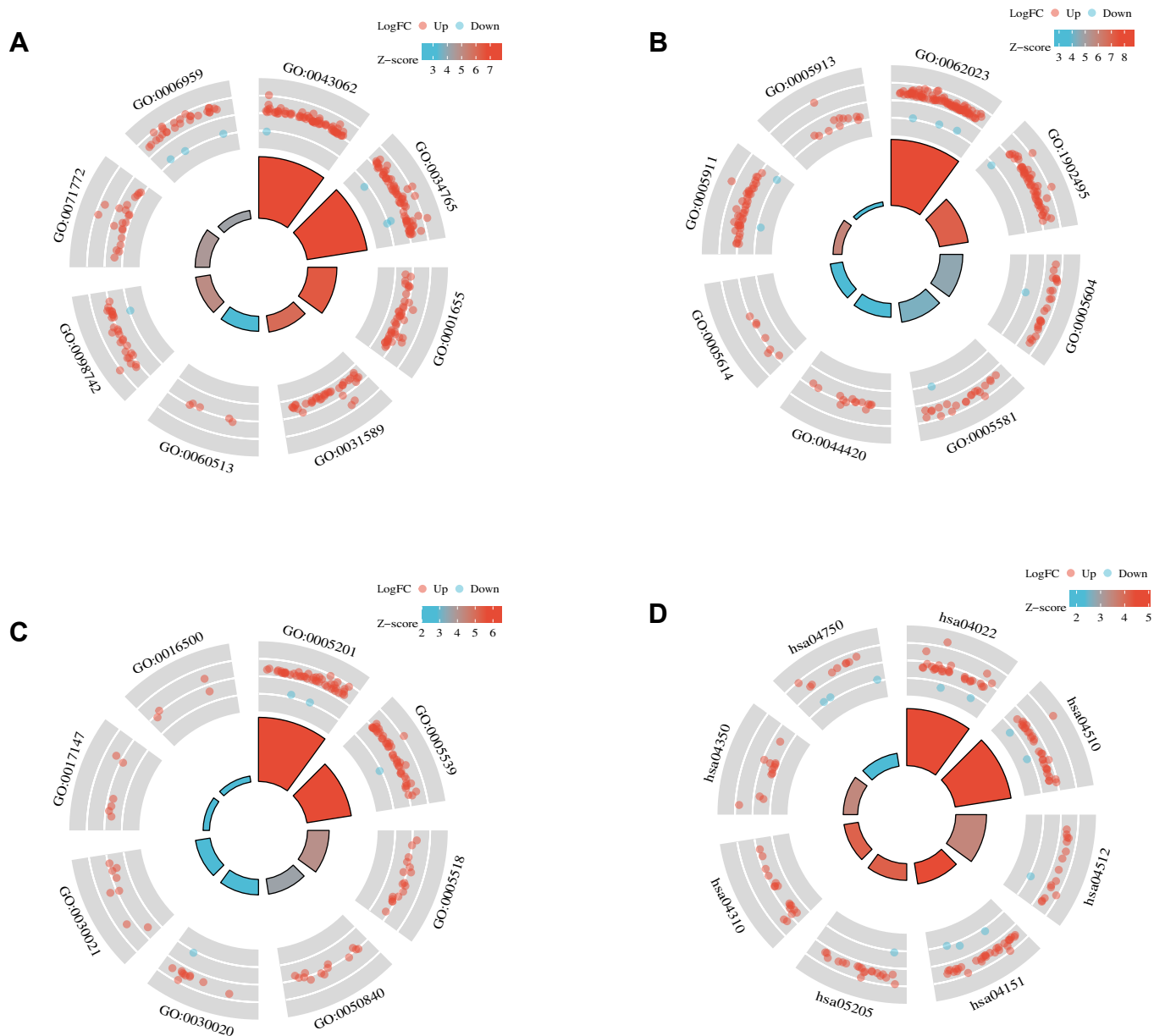

**Supplementary figure 5.** (A) BP, (B) CC, (C) MF and (D) KEGG pathways enriched.

Supplement: Supplementary file 6 [file DataSheet5.PDF]
